# 3D printing PCL/nHA bone scaffolds: exploring the influence of material synthesis techniques

**DOI:** 10.1186/s40824-021-00204-y

**Published:** 2021-01-26

**Authors:** Amanda Zimmerling, Zahra Yazdanpanah, David M. L. Cooper, James D. Johnston, Xiongbiao Chen

**Affiliations:** 1 Division of Biomedical Engineering, College of Engineering, University of Saskatchewan, Saskatoon, SK Canada; 2 Department of Anatomy Physiology and Pharmacology, College of Medicine, University of Saskatchewan, Saskatoon, Canada; 3 Department of Mechanical Engineering, College of Engineering, University of Saskatchewan, Saskatoon, SK Canada

**Keywords:** Three-dimensional printing, Bone scaffolds, Polycaprolactone, Nano-hydroxyapatite, Material synthesis

## Abstract

**Background:**

It is known that a number of parameters can influence the post-printing properties of bone tissue scaffolds. Previous research has primarily focused on the effect of parameters associated with scaffold design (e.g., scaffold porosity) and specific scaffold printing processes (e.g., printing pressure). To our knowledge, no studies have investigated variations in post-printing properties attributed to the techniques used to synthesize the materials for printing (e.g., melt-blending, powder blending, liquid solvent, and solid solvent).

**Methods:**

Four material preparation techniques were investigated to determine their influence on scaffold properties. Polycaprolactone/nano-hydroxyapatite 30% (wt.) materials were synthesized through melt-blending, powder blending, liquid solvent, and solid solvent techniques. The material printability and the properties of printed scaffolds, in terms of swelling/degradation, mechanical strength, morphology, and thermal properties, were examined and compared to one another using Kruskal-Wallis nonparametric statistical analysis.

**Results:**

Material prepared through the liquid solvent technique was found to have limited printability, while melt-blended material demonstrated the highest degree of uniformity and lowest extent of swelling and degradation. Scaffolds prepared with powder-blended material demonstrated the highest Young’s modulus, yield strength, and modulus of resilience; however, they also demonstrated the highest degree of variability. The higher degree of inhomogeneity in the material was further supported by thermal gravimetric analysis. While scaffolds printed from melt-blended, powder-blended, and solid solvent materials demonstrated a high degree of micro-porosity, the liquid solvent material preparation technique resulted in minimal micro-porosity.

**Conclusions:**

Study results indicate that specific techniques used to prepare materials influence the printing process and post-printing scaffold properties. Among the four techniques examined, melt-blended materials were found to be the most favorable, specifically when considering the combination of printability, consistent mechanical properties, and efficient preparation. Techniques determined to be favourable based on the properties investigated should undergo further studies related to biological properties and time-dependent properties beyond 21-days.

**Supplementary Information:**

The online version contains supplementary material available at 10.1186/s40824-021-00204-y.

## Background

Bone tissue engineering (BTE) is a growing field of study focussed on producing scaffolds for implantation into bone defect sites [1–3]. Ideal BTE scaffolds must be osteoinductive (cause pluripotent cells to differentiate into osteoblasts), osteoconductive (support ingrowth of capillaries and cells to form bone), biocompatible, biodegradable, and exhibit appropriate mechanical strength and biological properties [1, 3–6]. Factors currently known to influence the properties of BTE scaffolds mainly include scaffold design (e.g., porosity and materials used) and scaffold fabrication methods [7–9].

Interconnected pores approximately 300 μm in size promote vascularization, diffusion of nutrients, and cellular migration and attachment during tissue regeneration [7–10]. Three-dimensional (3D) printing technology allows for precise structural control and facilitates the strategic design and fabrication of complex structures featuring a high degree of porosity and pore interconnectivity [4, 8, 10–12]. In order to ensure the designed macro-porosity is present in printed scaffolds, the biomaterials used for fabrication must have a high degree of printability (extent to which the printed scaffold matches the CAD scaffold) [4, 13–15]. Biomaterial selection also influences swelling, degradation, and mechanical properties as materials vary in molecular weights, crystallinity, and surface chemistry [3–5]. Materials that exhibit sustained swelling often have increased hydrophilicity, which promotes cell attachment after implantation [6, 10, 16–19]. In BTE, degradation rate must be tailored to the specific injury to ensure that the scaffold does not degrade faster than the bone is able to regenerate to maintain proper mechanical support in the implant site [6, 13, 16, 20]. The mechanical properties of the scaffold should be similar to the mechanical properties of the natural bone at the defect site to avoid phenomena such as stress-shielding and ensure the implant is providing sufficient support [1, 21–23]. Biomaterial selection can also influence material micro-porosity, with greater micro-porosity leading to increased cellular attachment and proliferation [24].

Poly-ε-caprolactone (PCL) is a synthetic biodegradable polymer commonly used in BTE due to its ease of manipulation, biocompatibility, stability, and U.S. Food and Drug Administration (FDA) approval for use in some products [12, 16]; however, its hydrophobic and non-osteogenic nature decreases cell adhesion and bioactivity when implanted [7, 16]. The incorporation of nano-hydroxyapatite (nHA) to a PCL matrix is considered an effective approach to improve cell attachment by increasing hydrophilicity as well as improving mechanical properties, as shown in the literature [6, 20, 25–28]. As the major mineral component of bone, nHA is highly biocompatible, osteoconductive, and forms strong bonds with native bone, making it an excellent candidate for use in BTE [29].

While there has been substantial focus on the influence of scaffold design and fabrication technology, there has been little attention paid to how specific material preparation techniques influence the properties of printed scaffolds. Various material preparation techniques, such as melt-blending and liquid solvent, have been reported in literature for the preparation of PCL/nHA composite materials for printing bone scaffolds [10, 11, 17, 19, 27, 28, 30]. As material processing through use of heating or chemical solvents can affect material properties as well as the homogeneity of the fabricated material, the influence of material preparation techniques is important to the printing process and the properties of printed scaffolds. The present study aimed to compare the impact of material preparation techniques on the post-printing properties of printed scaffolds. Specifically, PCL/nHA (30% (wt.) nHA) composite materials were prepared via melt-blending, liquid solvent, solid solvent, and powder blending techniques, respectively, and then printed by extrusion printing. By maintaining consistency in scaffold design, raw biomaterials, and 3D printing technology, the effect of different material preparation technique was examined in terms of material printability, swelling/degradation, mechanical, morphological and thermal properties.

## Methods

### Materials

PCL pellets (M_w_ = 40,000–50,000, M_n_ = 45,000), nHA powder (particle size < 200 nm), and phosphate-buffered saline (PBS) pellets were purchased from Sigma Aldrich, Canada. PBS solution was made by dissolving 1 PBS pellet in 200 mL of distilled water. Stabilized methylene chloride (DCM) (purity ≥99.5%) was purchased from Fisher Scientific, Canada.

### Material preparation techniques

Various synthesis techniques were used to obtain PCL/nHA composites with 30% (wt.) nHA. This composition was selected due to favourable properties being reported in literature and results from a pilot study indicating sustained fluid uptake and improved mechanical properties when compared to pure PCL (Additional Figures 1 and 2) [11, 17, 19].

#### Melt-blending

PCL was melted in a beaker at 120 °C. nHA powder was added to the molten PCL and stirred to obtain a homogenous mixture. The material was left to cool before being cut into small pieces and stored at room temperature. Similar techniques have previously been reported in the literature for preparation of PCL/nHA composite materials [10, 30, 31].

#### Powder blending

PCL pellets were ground into a fine powder and mixed with nHA to form a homogeneous powder. The material was stored at room temperature until being loaded into the printing syringe. A similar method has been used in a related study [19].

#### Liquid solvent technique

PCL was dissolved in DCM and magnetically stirred at room temperature. nHA was added to the solution to form a slurry of PCL/nHA. The temperature was increased to 35 °C and the mixture was stirred vigorously to evaporate the solvent until a viscosity of 2.5 Pa•s was obtained, as measured by an Ultra Programmable Rheometer (Brookfield DV-III). The solution was then poured into the syringe for immediate printing. Liquid solvent techniques have previously been reported in literature [7, 32].

#### Solid solvent technique

Material was prepared using the same procedure as the liquid solvent technique; however, the solvent was fully evaporated. Once the material was too viscous to stir magnetically, it was left under a fume hood for 3 days to ensure full evaporation of the solvent. The solidified material was then cut into small pieces and stored at room temperature until printing. A similar technique has been reported in the literature [11].

### Scaffold design and printability

**Fig. 1:**
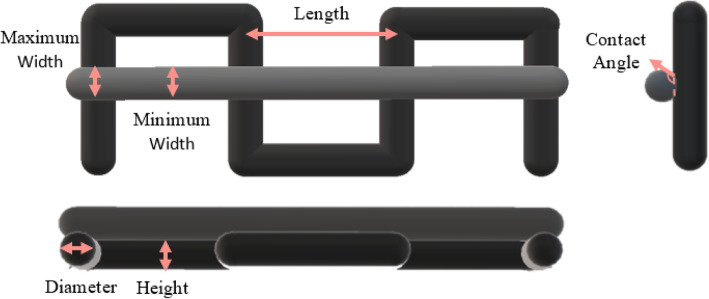
Schematic of dimensions of interest for scaffold printing

Scaffolds were designed using CAD software (Magics 13), where a strand diameter (*D*) of 0.510 mm and a strand spacing (*L*) of 1.0 mm were used as the design dimensions, providing an *L/D* ratio of 1.96. Layer height for printing was set as 80% of the strand diameter (0.408 mm) to account for gravitational spreading effects [13]. The theoretical contact angle (127**°)** was calculated using the set layer height and design diameter along with spherical cap relations. For the characterization of material printability, a two-layer structure was designed and fabricated as shown in Fig. 1. The high temperature head of a 3D Bioplotter Manufacturer Series system (EnvisionTEC GmbH) was outfitted with a cylindrical needle for all printing. Materials prepared through melt-blending, powder blending, and solid solvent techniques were printed at 120 °C onto a printing bed at a temperature of 35 °C. The material prepared using the liquid solvent technique was printed at 30 °C onto a printing bed at a temperature of 15 °C to limit spreading. Two-layer scaffolds composed of PCL/nHA 30% prepared by the different techniques were printed as the printing speed and pressure were varied to obtain printed strands analogous to the designed strands (*n* = 10 per material preparation technique) [13]. The scaffolds were then imaged under an optical microscope (Leica DMIL) at 100x magnification. ImageJ software was used to compare the printed scaffold dimensions to the design dimensions to quantitatively characterize the printability of the various materials through assessment of discrepancies between the CAD model and the printed scaffold [33].

The designed and theoretically calculated scaffold dimensions were compared to the measured dimensions to determine the material printability, with greater adherence to the designed dimensions indicating a high degree of printability. The printing pressure and speed that produced scaffolds most comparable to the design were used to fabricate scaffolds for subsequent tests. This approach limited differences in overall macro-porosity, pore size, pore shape, and pore interconnectivity between scaffolds prepared via the different synthesis techniques.

### Degradation and swelling

Eight two-layer scaffolds of each material were immersed in PBS medium and incubated at 37 °C with 5% CO_2_. The PBS solution was refreshed every 3 days to ensure maintained activity. Two scaffolds of each material were removed at time points of 3, 7, 14, and 21 days. The removed scaffolds were blotted dry to remove excess medium before being weighed in the wet condition. Swelling was calculated using Eq. (1).

1 $$ Swelling\ \left(\%\right)=\frac{M_w-{M}_i}{M_i}\times 100 $$

where *M*_*w*_, and *M*_*i*_ are the wet mass and the initial mass, respectively.

The scaffolds were then fully dried under vacuum to remove residual PBS before being weighed to determine mass loss using Eq. (2), where *M*_*f*_ is final mass. The average values from the scaffolds were calculated and recorded.

2 $$ Mass\ Loss\ \left(\%\right)=\frac{M_i-{M}_f}{M_i}\times 100 $$

### Mechanical testing

Ten-layer (12 mm × 12 mm × 4 mm) PCL/nHA 30% scaffold specimens were fabricated based on the CAD model using material prepared through melt-blending, powder blending, and solid and liquid solvent techniques. Three scaffolds of each material underwent compression testing using a material testing system (MTS Bionix® Servohydraulic Test System) with a load cell of 5.0 kN and a crosshead speed of 1.0 mm/min. Apparent compressive modulus (*E*) and yield strength (*S*_*y*_) were derived from the stress-strain curve, while modulus of resilience was calculated using Eq. (3).

3 $$ U={\left({S}_y\right)}^2/2E $$

### Scanning Electron microscopy (SEM)

A Hitachi SU8010 SEM was used to characterize surface morphology and microstructure. Two-layer composite scaffolds prepared via the four different material preparation techniques were coated with 10 nm of gold using a Quorum Q150TES Sputter Coater, and mounted in the specimen holder with double-sided tape. All the samples were scanned at an accelerating voltage of 3.0 kV with magnifications ranging from 30x to 1500x. Micro-porosity, in this instance defined as pores within the material strands themselves, were analyzed with respect to both depth and number.

### Thermal gravimetric analysis (TGA)

Thermal gravimetric analysis of scaffolds prepared through melt-blending, powder blending, liquid solvent and solid solvent techniques were obtained using a TA Instruments Q50 V20. The analysis temperature was increased from 20 to 500 °C at a rate of 10 °C/min. This ensured the decomposition of PCL and allowed for the experimental mass of nHA in the scaffold to be measured and thermal stability of the materials to be assessed.

### Statistical analysis

One sample t-tests were used to compare measured dimensions of the scaffolds fabricated using different preparation techniques to the CAD model dimensions. A significance value of *p* < 0.05 was used to indicate that the scaffolds of a specific material preparation group deviated from the CAD dimension. Coefficient of variation (*CV%)*, used to assess variation in mechanical properties and thermogravimetric analysis results, was calculated as shown in Eq. (4).

4 $$ \mathrm{CV}\%=\mathrm{SD}/\mathrm{Mean} $$

Two samples of each material preparation technique were measured for TGA and at each time point for swelling/degradation tests. Three samples were tested for mechanical properties. The effect of different synthesis methods on swelling and degradation was compared at each specific time point. Analysis of swelling and degradation over time was also evaluated for each individual synthesis method.

GraphPad Prism 8 statistical analysis software was used to complete non-parametric Kruskal-Wallis tests due to the small sample sizes being analyzed. If an overall significant difference between groups was observed, post-hoc Dunn’s test was used for pairwise comparisons. All graphs are presented based on the “min to max, show all points” method, through box-and-whisker plots with each individual value indicated on the graph.

## Results

### Printability

**Fig. 2:**
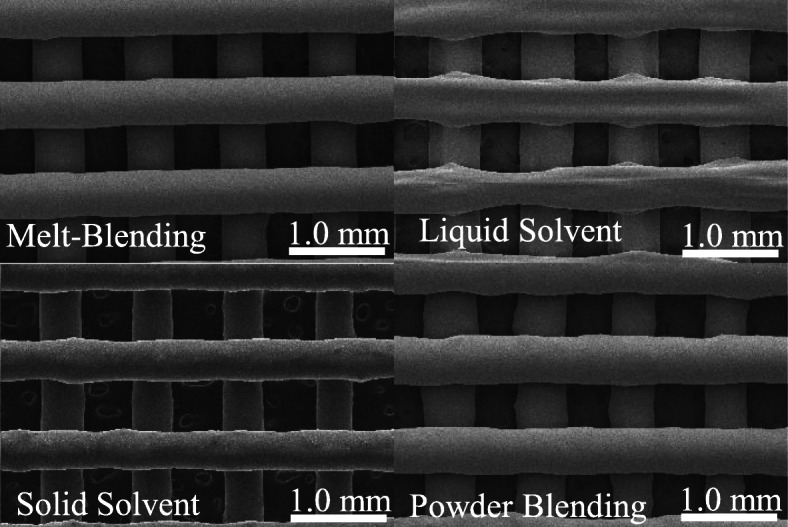
SEM image of optimized 3D printed scaffolds (30X magnification) for various material preparation techniques

Well-defined and consistent macro-porosity was obtained within all materials; however, scaffolds fabricated from melt-blended material showed a relatively uniform strand diameter while other material preparation techniques resulted in scaffolds with less uniformity (Fig. 2, Table 1).

**Table 1:** Dimensions of optimized scaffolds for different material preparation techniques of PCL/nHA reported as mean ± SD (* indicates *p* < 0.05 when compared to the theoretical CAD value)

Preparation Technique	Diameter (mm)	L/D Ratio	Height (mm)	Contact Angle (°)	Uniformity
Theoretical	0.510	1.96	0.408	127	1.000
Melt-Blending	0.515 ± 0.037	1.92 ± 0.15	0.456 ± 0.030*	89 ± 14*	0.949 ± 0.117
Liquid Solvent	0.544 ± 0.064	1.88 ± 0.25	0.084 ± 0.022*	13 ± 3*	0.875 ± 0.157*
Solid Solvent	0.478 ± 0.062	2.13 ± 0.31	0.413 ± 0.039	73 ± 4*	0.880 ± 0.146*
Powder Blending	0.517 ± 0.035	1.96 ± 0.16	0.390 ± 0.052	107 ± 11	0.873 ± 0.094*

Material prepared using the liquid solvent technique demonstrated the largest discrepancies with the CAD modeled dimensions as it lacked structural integrity upon printing, leading to a height reduction of ~ 80% (*p* = 0.002) and a contact angle reduction of ~ 90% (*p* < 0.001). As such, the liquid solvent material required a reduced layer height (0.100 mm) to print multi-layer scaffolds and was considered to have poor printability.

### Swelling and degradation

**Fig. 3:**
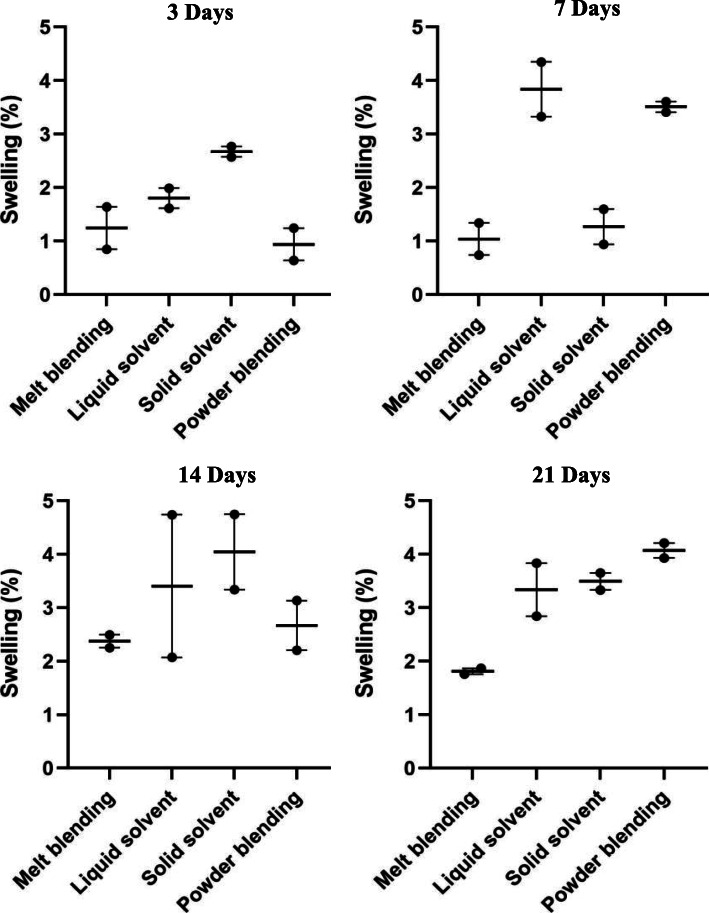
Swelling (%) of scaffolds printed using different material preparation techniques at various time points

**Fig. 4:**
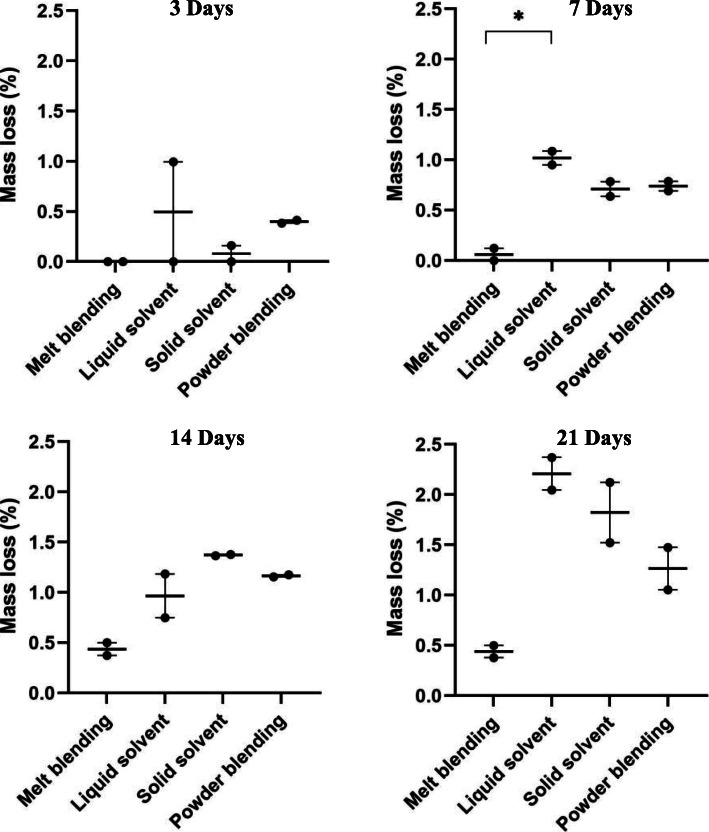
Mass loss (%) of scaffolds made using different material preparation techniques at various time points

Swelling and degradation results were compared both within material preparation techniques over time (Additional Figures 3 and 4) and between material preparation techniques at each measurement interval (Figs. 3 and 4). All PCL/nHA 30% scaffolds maintained or increased their water uptake over the 21-day immersion period; however, none of the material preparation techniques demonstrated a significant difference when swelling at time points ranging from 3-days to 21-days were compared using non-parametric Kruskal-Wallis statistical analysis (*p* < 0.05). There was also no statistically significant difference in swelling between material preparation techniques when they were compared at each time point (Fig. 3). A general trend of less swelling in the melt-blended material can be seen at time points past the 3-day mark.

All materials demonstrated increased mass loss from initial immersion to the final 21-day time point for on-going degradation; however, none of the time-dependent mass losses were statistically different. Compared to melt-blending and solid solvent, scaffolds fabricated with liquid solvent and powder blended materials demonstrated relatively larger mass losses after 3 days immersion (Fig. 4), likely due to further solvent evaporation (liquid solvent technique) and detachment of loosely adhered nHA powder (powder blending).

When comparing degradation between material preparation techniques, the only significant difference was found at the 7-day time point between melt-blending and liquid solvent materials (*p < 0.05*); however, a general trend of less degradation of the melt-blended material can be seen at each time point.

### Mechanical testing

Due to the poor printability of material prepared through the liquid solvent technique, the required height for mechanical testing was unable to be fabricated and thus mechanical testing results of these scaffolds are not reported.

**Fig. 5:**
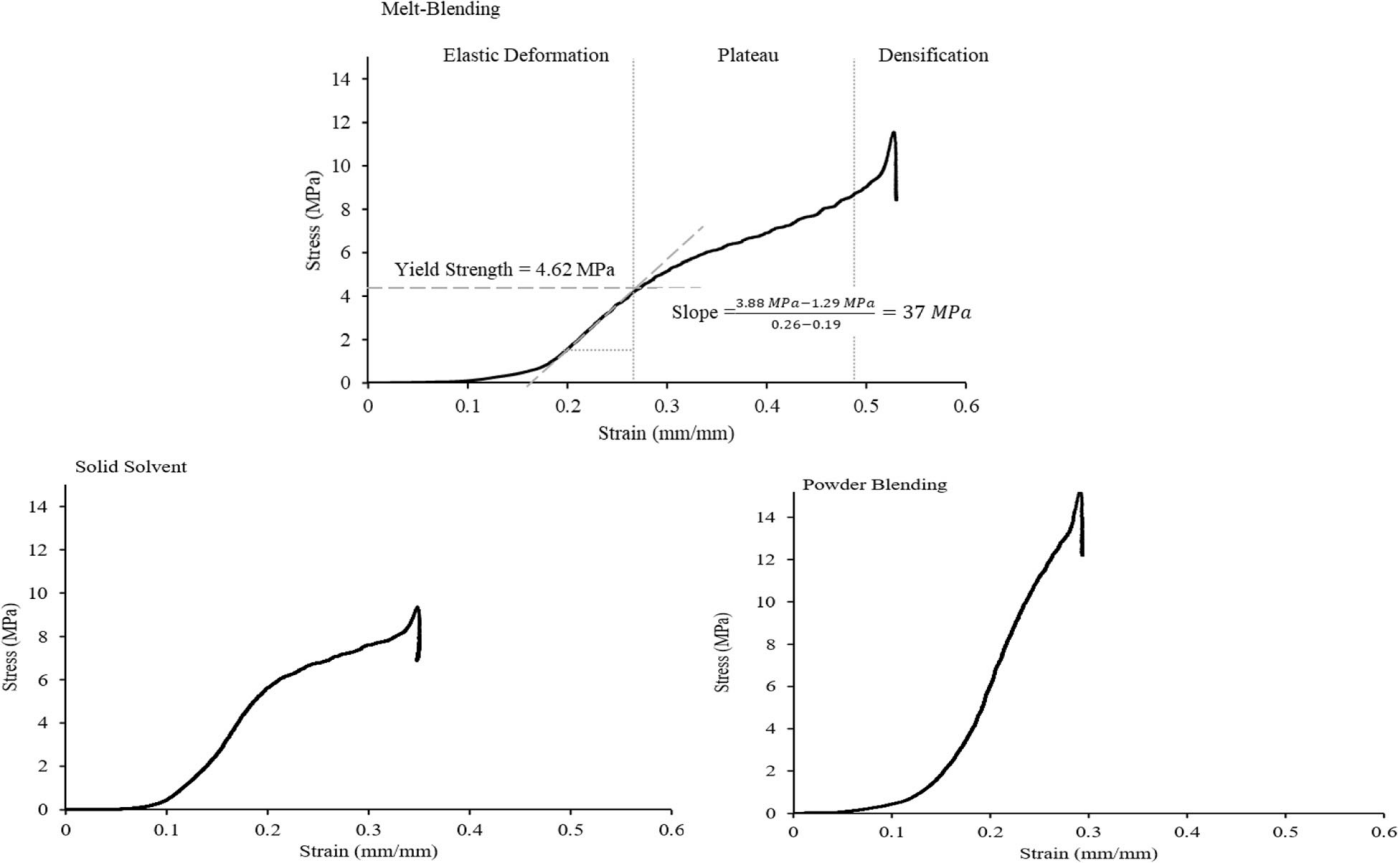
Representative stress-strain plots for scaffolds fabricated with materials prepared through various synthesis techniques

Figure 5 displays the stress-strain curves obtained from compressive testing of scaffolds prepared with melt-blended, solid solvent, and powder blended materials. An initial linear region demonstrates the elastic deformation of the scaffolds, followed by a plateau region indicating plastic deformation of the scaffold structure. This region is followed by an increase in slope representing the on-set of densification. This indicates failure of the scaffold structure as pores are fully compressed. At this stage, any further testing represents the compressive strength of bulk material and was not considered in analysis. From these plots, it is evident that scaffolds prepared via powder blending had a limited plateau region, indicating brittle behaviour with limited ductility.

Scaffolds made from powder blended material showed the highest compressive (Young’s) modulus, yield strength and modulus of resilience (Table 2). Melt-blending and solid solvent techniques offered similar yield strengths with slight differences in compressive moduli and moduli of resilience.

**Table 2:** Compressive strength, compressive modulus and modulus of resilience of scaffolds prepared using different material preparation techniques of PCL/nHA 30% reported as mean ± SD (CV%)

Preparation Technique	Yield Strength (MPa)	Compressive Modulus (MPa)	Modulus of Resilience
Melt-Blending	4.81 ± 0.24 (5.0%)	39.71 ± 3.38 (8.5%)	0.29 ± 0.03 (10%)
Liquid Solvent	N/A	N/A	NA
Solid Solvent	4.71 ± 0.43 (9.1%)	57.81 ± 1.96 (3.4%)	0.19 ± 0.02 (11%)
Powder Blending	10.40 ± 1.39 (13%)	116.73 ± 45.23 (39%)	0.46 ± 0.19 (41%)

**Fig. 6:**
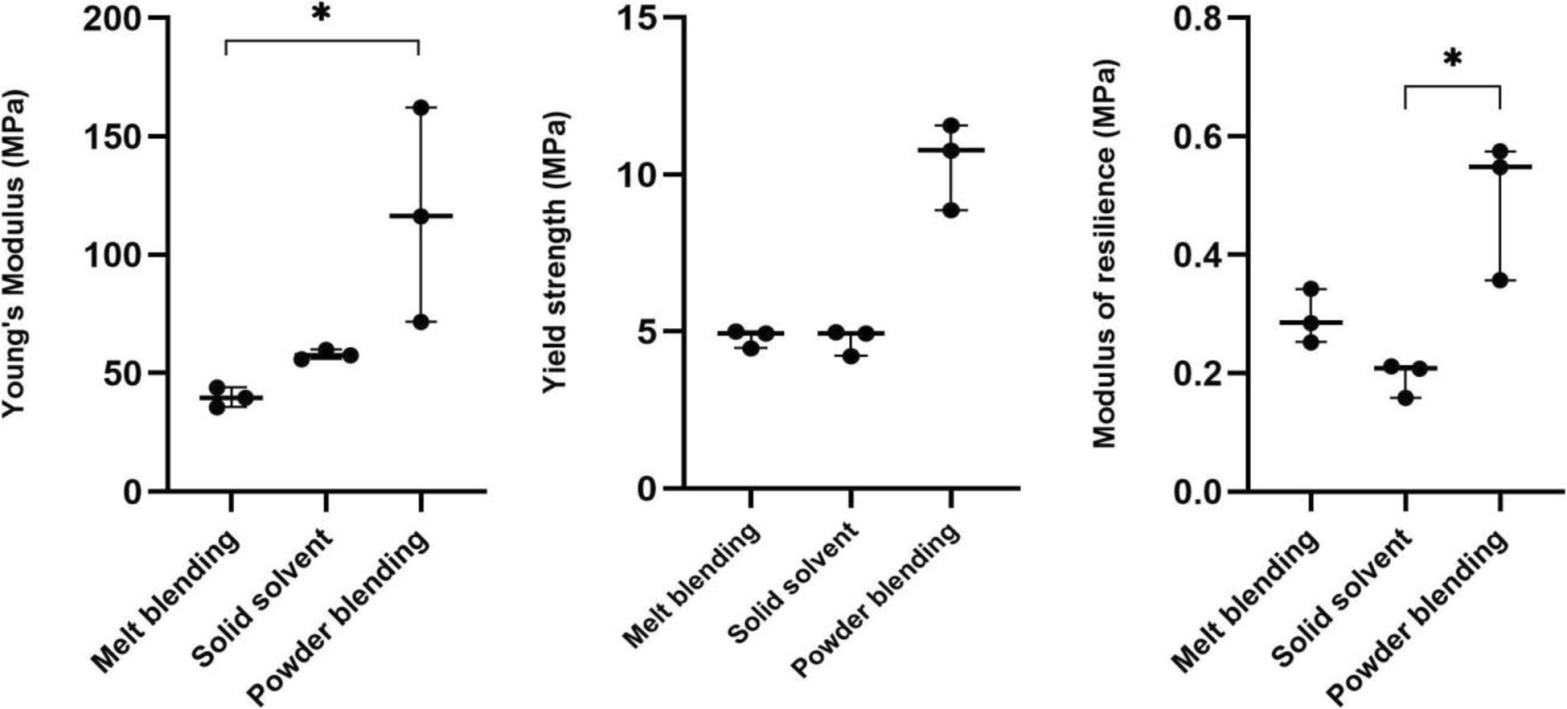
Young’s modulus, yield strength and modulus of resilience of scaffolds prepared using different preparation techniques

Statistical analyses of these results indicated that there was a significant difference in Young’s modulus between melt-blending and powder blending, as well as a significant difference in modulus of resilience between solid solvent and powder blending (Fig. 6).

Although statistically significant differences were not noted, higher mechanical strength of scaffolds prepared through the powder blending method is evident; however, large variation (assessed via CV%) in the mechanical properties of powder blended scaffolds is also evident while melt-blended and solid solvent material demonstrated minimal variance in mechanical properties.

### SEM

**Fig. 7:**
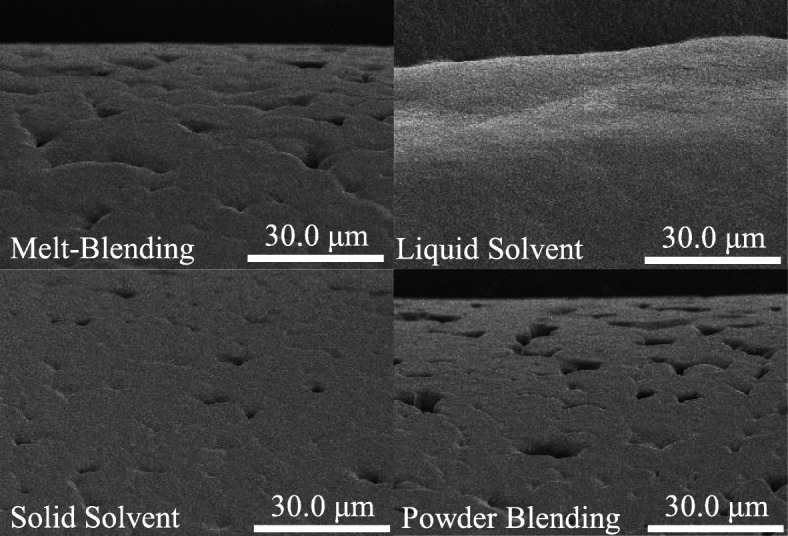
SEM images at 1500X magnification of scaffolds printed using materials prepared by various synthesis techniques

As shown in Fig. 7, micro-pores were visible in the scaffolds using melt-blended, solid solvent and powder blended materials, while no micro-pores were evident on the surface of the liquid solvent material scaffolds. The powder blended scaffold showed the deepest and highest number of pores on the strand surface. The smooth surface of the liquid solvent-based material scaffold was likely due to the lower viscosity of the liquid, which allowed for the material to fill pores during printing.

### TGA

Thermogravimetric analysis was used to assess the homogeneity of the materials prepared through various techniques, as well as the thermal stability of the materials. All synthesis techniques produced material with experimental compositions close to the design value of 30% nHA (Table 3). Kruskal-Wallis statistical analysis indicated no differences between the synthesis techniques (*p* = 0.84). This analysis also demonstrated that the powder blending material preparation method was unable to match the compositional consistency of the melt-blending and solid solvent techniques (CV% = 21% compared to 0.41 and 0.70%, respectively).

**Table 3:** Experimental nHA composition of scaffolds made with materials prepared through the various material preparation techniques reported as mean (CV%)

Preparation Method	Experimental nHA Composition
Melt-Blending	29.60 (0.41%)
Liquid Solvent Technique	29.98 (4.3%)
Solid Solvent Technique	30.02 (0.70%)
Powder Blending	26.77 (21%)

TGA was also used to determine if the material preparation method had any effect on the thermal stability of the material created. All of the scaffolds demonstrated a one-step decomposition profile with a single transition temperature and a similar thermal stability, with decomposition starting around 250 °C; however, the scaffolds printed with melt-blended material appeared to finish degradation at a higher temperature compared to the other scaffolds (375 °C compared to 340 °C, respectively). This may be due to the extended time that melt-blended materials spend at high temperature during processing.

## Discussion

This exploratory study indicates that the material preparation technique used in the creation of material later used to fabricate tissue scaffolds may impact post-printing properties of the fabricated scaffold. With raw biomaterial, scaffold design and printing technology held constant, printability analysis showed minor differences between the solid solvent, powder blended, and melt-blended materials, with the melt-blended material demonstrating a slightly greater degree of printability and the liquid solvent material demonstrating decreased printability. As material prepared through the liquid solvent technique relies on solvent evaporation instead of cooling to solidify, the solidification process for this material was more time-consuming leading to reduced shape fidelity. Initiating printing with material prepared through the liquid solvent technique after it has reached a higher viscosity may increase printability; however, the material viscosity was also found to vary over the printing time, with printing parameters requiring adjustment to ensure consistent structures. Due to this, it was concluded that the liquid solvent material preparation technique provided material with poor printability. Optimization carried out during the printability analysis successfully allowed for the fabrication of scaffolds with similar pore size, pore shape, and interconnectivity.

As macro-porosity of the scaffolds was held consistent, variation in swelling and degradation is likely due to differences in micro-porosity or changes in material properties associated with specific material preparation techniques. Scaffolds exhibiting greater micro-porosity would be expected to demonstrate increased swelling and degradation due to a larger surface area for interaction with the medium. As melt-blended PCL/nHA 30% materials (which demonstrated the least swelling) have been reported to have satisfactory biological compatibility, and given that increased swelling commonly signifies increased hydrophilicity, it is expected that all of the material preparation techniques would demonstrate satisfactory biological compatibility for use in BTE scaffolds [10, 16, 19, 29]. Melt-blended scaffolds demonstrated significantly less mass loss than liquid solvent scaffolds at the time point of 7 days and, in general, demonstrated less mass loss when compared to materials prepared through other methods. Some of the increased mass loss incurred by non-melt blended scaffolds can be attributed to greater immersed surface area resulting from less uniform strands [9, 34]. Study results indicated an increase in mass loss over time, which is consistent with reports in the literature [34–36]. This provides support to the general trends indicated by experimental results and demonstrates the need for further studies assessing swelling and degradation characteristics of various material preparation techniques over a longer time frame. These specific degradation studies were selected as they provide information regarding relative degradation rates between the various materials without biological influences, as commonly reported in the literature [34–36]. The recorded rates represent passive degradation with variation due to mechanical differences in the materials. In vivo degradation studies are required to analyze the relationship between bone regeneration and scaffold degradation. As the materials’ biological compatibility will influence the degradation rates observed in these studies [36], it is important to consider passive and active degradation separately.

Although scaffolds fabricated using the powder blending technique demonstrated the largest mechanical properties, there was a large variance in the experimental values obtained, likely due to inhomogeneity in the material. In order to quantify the variance of results obtained from each material, the coefficient of variation was calculated. Solid solvent and melt-blended materials demonstrated a high degree of consistency in yield strength (CV% of 5.0 and 9.1%, respectively) and compressive modulus (CV% of 8.5 and 3.4%, respectively), leading to the conclusion that materials prepared through these techniques are homogeneous, while powder blended scaffolds are less homogenous (as indicated by CV% of 13 and 39% for yield strength and compressive modulus, respectively). Due to the inconsistency in scaffolds prepared with powder blended material, it is considered less suitable for fabrication of mechanically consistent bone tissue scaffolds. The lack of structural fidelity of liquid solvent scaffolds observed during printability analysis, and the inability to fabricate scaffolds tall enough for mechanical testing, demonstrates that liquid solvent material preparation techniques described here are also unsuitable for BTE scaffold fabrication.

Morphological analysis indicated differences caused by material preparation technique as the strand surface from the liquid solvent technique demonstrated minimal micro-porosity when compared to other preparation techniques. Micro-pores on the surface of the solid solvent and melt blended material scaffolds were likely formed during cooling or by air trapped in the material during melting in the high temperature printing head [9, 17, 37]. The powder blended material appeared to demonstrate slightly deeper micro-pores, likely due to a lack of homogeneity during mixing leading to aggregated nHA powder that lacked adherence to the PCL. The dislodgement of this powder could then lead to deeper and more numerous micro-pores than what would be found in homogeneous materials. Thermal analysis indicated that all materials had the desired composition as there were no statistically significant differences from the desired value with minimal deviations in thermal stability. Of note, as the glass transition temperature of PCL is ~ 60 °C, glass transition was assumed to have no major influence on the properties of the PCL/nHA scaffolds.

Differences due to material preparation technique demonstrated in this study may be due to many factors. The melt-blending technique requires mixing of raw material at elevated temperature for extended periods of time while the solid and liquid solvent techniques involve interactions of the raw materials (PCL and nHA) with an organic solvent (DCM). These thermal and chemical interactions may cause lasting changes in crystallinity and extent of polymer chain entanglement. The powder blending procedure relies upon the melting and extrusion processes of the extrusion printer to incorporate nHA particles into the melted PCL, which led to varying concentrations of nHA throughout the fabricated scaffolds. This may explain the variation exhibited by powder blended scaffolds in this study.

Overall, a limitation to this exploratory study was small sample sizes (and associated low statistical power) which may account, in part, for the lack of observed differences between the preparation techniques; however, general trends in the data generated, and the existence of different trends between scaffolds fabricated from material prepared using various techniques demonstrates that material preparation technique does influence the post-printing properties of the scaffold. More in-depth study of these influences, including higher powered analysis of swelling/degradation and mechanical properties would be beneficial in selecting an optimal technique; however, melt-blending and solid solvent preparation techniques appear to be best suited for application in BTE as they demonstrated a high degree of printability, consistent mechanical properties, and beneficial micro-porosity. Material prepared through the liquid solvent technique failed to maintain shape fidelity and demonstrated no micro-porosity while material prepared through powder blending was not homogeneous and showed large variation in mechanical and compositional properties. Melt-blending was a less time-consuming preparation technique compared to the solid solvent technique as it did not require time for solvent evaporation, and it also demonstrated a higher thermal stability when compared to other techniques. Though, it demonstrated less swelling than material prepared through the solid solvent technique. Along with higher-powered studies, melt-blending and solid solvent material preparation techniques should undergo a biocompatibility study to further distinguish the post-printing properties obtained using materials prepared through these two techniques. This is needed as the solvent used in preparing the solid solvent material may have a residual affect on cellular compatibility [13, 37]. As melt-blending is the more efficient preparation technique, it is favourable bearing the findings of biological compatibility studies. Importantly, the results of this exploratory study can guide future cellular and in vivo studies required for biological analysis using favorable approaches identified here.

## Conclusions

Synthesis of materials that occur pre-fabrication is important to the printing process and the post-printing properties of scaffolds. This study aimed to determine the influence of material synthesis techniques on the post-printing properties of fabricated scaffolds in terms of printability, swelling, degradation, mechanical, morphological, and thermal properties. PCL/nHA 30% (wt.) material was successfully synthesized through four preparation techniques, specifically melt-blending, powder blending, liquid solvent, and solid solvent techniques. Printability assessments determined that material prepared through the liquid solvent technique demonstrated limited printability due to its reduced rate of solidification, while melt-blending demonstrated the highest degree of printability closely conforming to the CAD model dimensions. Swelling and degradation analysis determined that melt-blended material demonstrated reduced swelling and degradation compared to the other material synthesis techniques. Scaffolds fabricated with powder-blended material demonstrated the largest Young’s modulus, yield strength and modulus of resilience; however, the scaffolds also demonstrated the greatest variability, indicating that scaffolds prepared with powder-blended material were inhomogeneous. This finding was further supported by increased variability in composition in powder-blended material found via TGA. The morphology was also influenced by material preparation technique, as liquid solvent materials demonstrated no micro-porosity in comparison to other material preparation techniques. From this exploratory study, melt-blending is indicated as a favourable material preparation technique as it is an efficient method for production of material and material prepared in this way demonstrates a high-degree of printability, consistent mechanical properties and slow degradation rates. Taken together, this study illustrates that material preparation is of importance in printing bone tissue scaffolds in terms of material printability and various physical properties.

## Supplementary Information

**Additional file 1.**

**Additional file 2.**

**Additional file 3.**

**Additional file 4.**

## Data Availability

The datasets used and/or analysed during the current study are available from the corresponding author on reasonable request.

## Abbreviations

BTE: Bone tissue engineering
3D: Three-dimensional
CAD: Computer aided design
PCL: Poly-ε-caprolactone
FDA: U.S. Food and Drug Administration
nHA: Nano-hydroxyapatite
PBS: Phosphate-buffered saline
DCM: Methylene chloride
SEM: Scanning electron microscopy
TGA: Thermal gravimetric analysis
CV%: Coefficient of variation
SD: Standard deviation
