# Supplementary material for: 3D printing PCL/nHA bone scaffolds: exploring the influence of material synthesis techniques
Source: Biomater Res. 2021 Jan 26;25:3. doi: 10.1186/s40824-021-00204-y (PMC7836567; doi:10.1186/s40824-021-00204-y)
Supplement: Supplementary file 1 — Additional file 1. [file 40824_2021_204_MOESM1_ESM.pptx]

## Slide 1
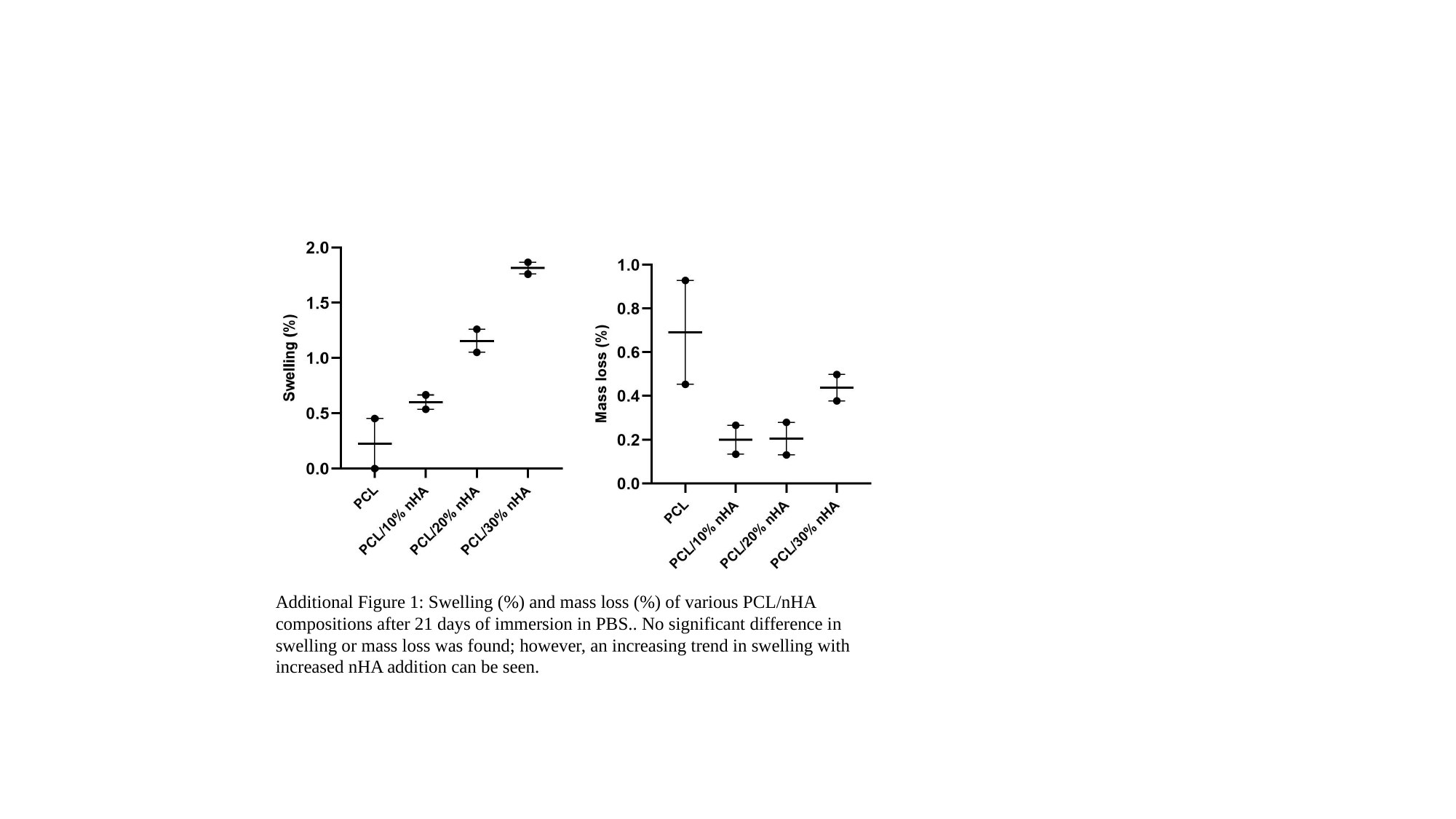

Additional Figure 1: Swelling (%) and mass loss (%) of various PCL/nHA compositions after 21 days of immersion in PBS.. No significant difference in swelling or mass loss was found; however, an increasing trend in swelling with increased nHA addition can be seen.
