# Supplementary material for: 3D printing PCL/nHA bone scaffolds: exploring the influence of material synthesis techniques
Source: Biomater Res. 2021 Jan 26;25:3. doi: 10.1186/s40824-021-00204-y (PMC7836567; doi:10.1186/s40824-021-00204-y)
Supplement: Supplementary file 2 — Additional file 2. [file 40824_2021_204_MOESM2_ESM.pptx]

## Slide 1
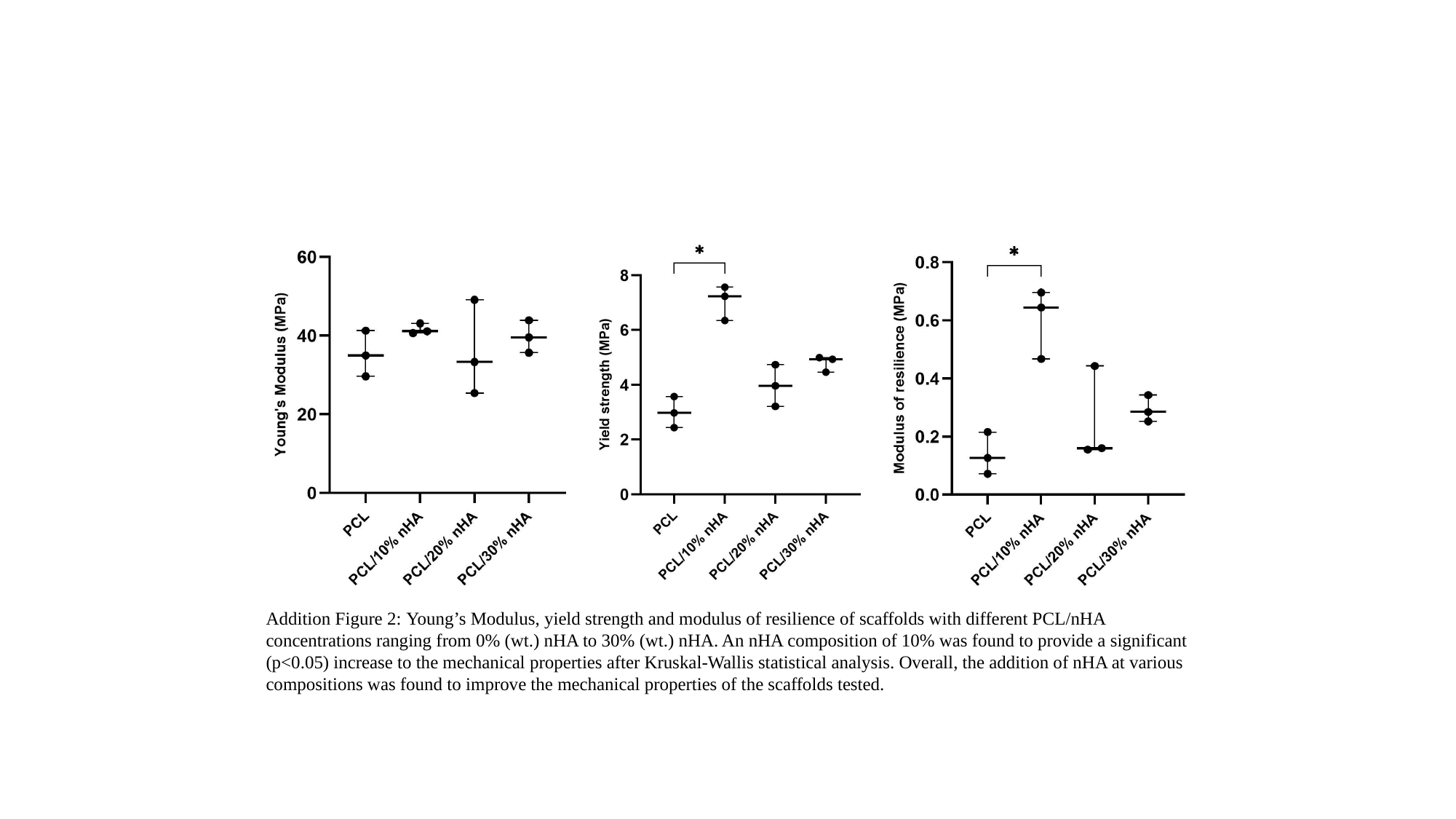

Addition Figure 2: Young’s Modulus, yield strength and modulus of resilience of scaffolds with different PCL/nHA concentrations ranging from 0% (wt.) nHA to 30% (wt.) nHA. An nHA composition of 10% was found to provide a significant (p<0.05) increase to the mechanical properties after Kruskal-Wallis statistical analysis. Overall, the addition of nHA at various compositions was found to improve the mechanical properties of the scaffolds tested.
