# Supplementary material for: 3D printing PCL/nHA bone scaffolds: exploring the influence of material synthesis techniques
Source: Biomater Res. 2021 Jan 26;25:3. doi: 10.1186/s40824-021-00204-y (PMC7836567; doi:10.1186/s40824-021-00204-y)
Supplement: Supplementary file 4 — Additional file 4. [file 40824_2021_204_MOESM4_ESM.pptx]

## Slide 1
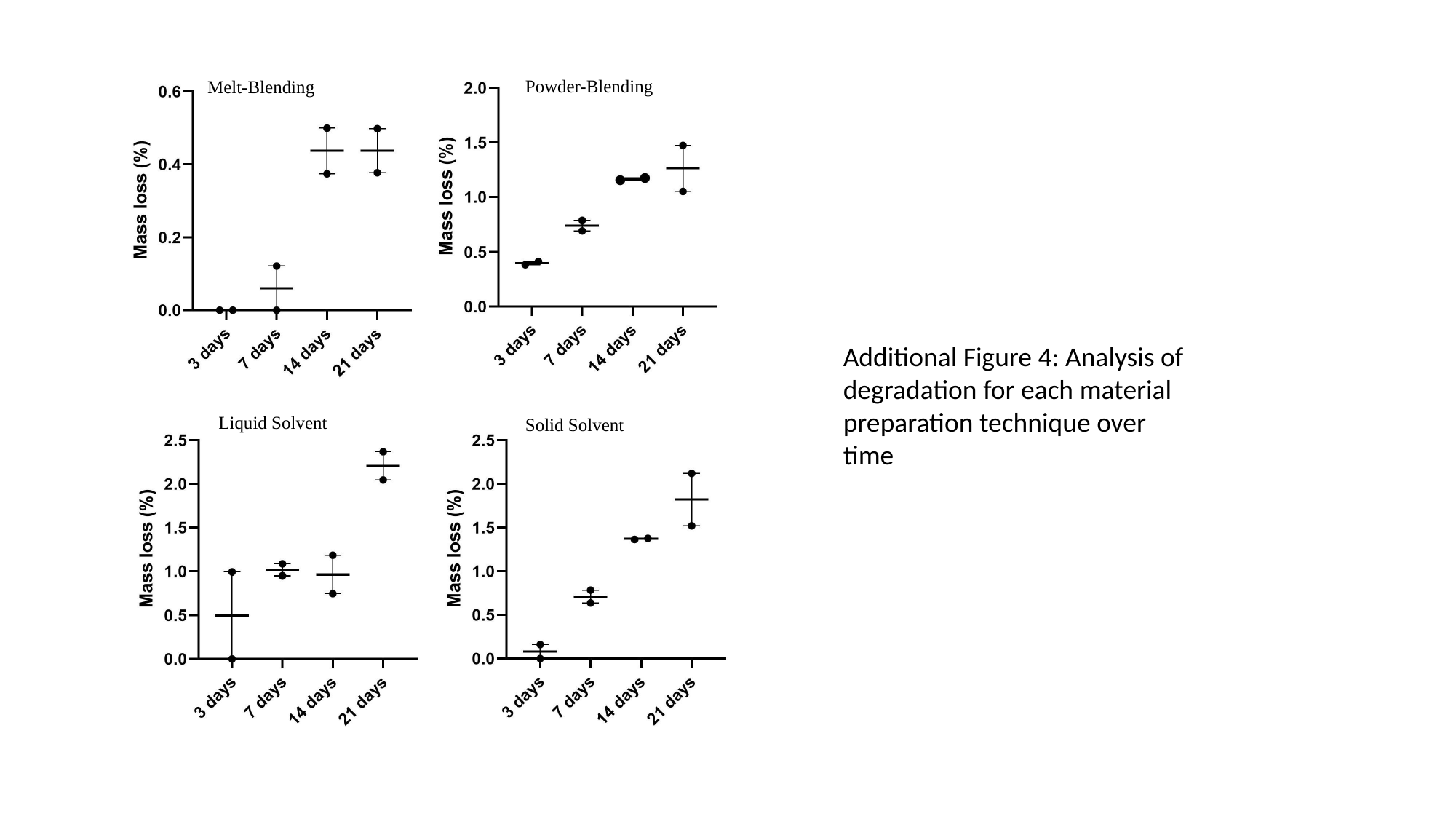

Powder-Blending
Melt-Blending
Liquid Solvent
Solid Solvent
Additional Figure 4: Analysis of degradation for each material preparation technique over time
